# Abdominal defect reconstruction due to advanced lobomycosis with anterolateral thigh (alt) flap: a case report

**DOI:** 10.1080/23320885.2026.2655578

**Published:** 2026-04-06

**Authors:** Frank Andrés Álvarez Vásquez, Iván Enrique Rodríguez Mantilla, Nicol Daniella Cala Gómez

**Affiliations:** a Universidad del Rosario. Epidemiologist – Universidad Autónoma de Bucaramanga; b Fundación Universitaria de Ciencias de la Salud. Fellowship in Advanced Oncologic Plastic Surgery, Reconstructive Microsurgery, and Lymphedema Surgery, Taichung, Taiwan; c Universidad Nacional de Colombia

**Keywords:** Lobomycosis, mycoses, skin diseases, surgical flaps, reconstructive surgical procedures, abdominal wall

## Abstract

Lobomycosis, also known as lacaziosis, is a chronic subcutaneous mycosis caused by *Lacazia loboi* and endemic to tropical regions of South America. Medical therapy alone has demonstrated limited efficacy, and wide surgical excision remains the mainstay of treatment, particularly in extensive or long-standing disease. However, radical resection may result in complex soft tissue defects requiring advanced reconstructive strategies. We report the case of a 42-year-old male with advanced lobomycosis involving the left lower hemiabdomen. Wide local excision with 2 cm macroscopic margins was performed, resulting in exposure of the anterior rectus sheath and external oblique aponeurosis. Immediate reconstruction was achieved using a pedicled anterolateral thigh (ALT) flap based on musculocutaneous perforators of the descending branch of the lateral circumflex femoral artery. The flap was rotated 180 degrees to increase its arc of reach and allow tension-free inset without microvascular anastomosis. The donor site was managed with split-thickness skin grafting. Postoperatively, adjuvant systemic itraconazole therapy was administered. The flap demonstrated complete viability with no vascular compromise, and satisfactory functional and aesthetic outcomes were achieved. This case highlights the pedicled ALT flap as a safe, versatile, and reliable reconstructive option for extensive abdominal defects secondary to deep mycoses, offering robust vascularity and low donor-site morbidity while preserving abdominal wall function.

## Introduction

Lobomycosis (LM), also known as lacaziosis or Jorge Lobo’s disease, is a chronic subcutaneous mycosis caused by the yeast-like fungus *Lacazia loboi* (LL) [1,2]. It was first described in 1931 by the Brazilian dermatologist Jorge Lobo in the Amazon region of Brazil [3–5]. Since then, multiple cases have been reported in South American countries, particularly in tropical and subtropical areas where environmental conditions favor the persistence and transmission of the fungus [6,7].

This keloid-like blastomycosis is characterized by the appearance of slowly growing, chronic cutaneous lesions, adopting nodulo-keloidal or verrucous forms, most commonly located on the extremities, trunk, or auricular pinnae [8,9]. It predominantly affects young men and adults between 20 and 40 years of age, whose occupational or lifestyle activities involve continuous exposure to the rainforest environment, such as farmers, hunters, loggers, and members of indigenous communities [10,11].

Although its overall incidence is low—hindering the collection of detailed nationwide statistics—cases of LM have been documented in Colombia in various regions, including the Amazon, Orinoquía, and the Pacific coast, with the Amazonian region accounting for the highest number of reports [8]. This geographical distribution highlights the importance of clinical recognition of LM in at-risk populations.

The differential diagnosis includes other chronic granulomatous dermatoses such as leishmaniasis, leprosy, and keloids [12,13]. The definitive diagnosis is established through histopathological examination, which reveals dermal granulomas containing rounded yeast cells of LL arranged in chains, displaying a characteristic ‘string of pearls’ or ‘rosary-like’ morphology. These fungal structures are best visualized with special stains such as periodic acid–Schiff (PAS) or Grocott’s methenamine silver (GMS) [14–16].

Medical treatment has shown limited efficacy due to the marked resistance of LL to most conventional antifungal agents, including itraconazole and amphotericin B [17]. In this context, complete surgical excision with wide safety margins has become the treatment of choice, especially in localized or recurrent cases of LM, as incomplete resection has been associated with high recurrence rates reported in the literature. Nevertheless, when surgical excision results in large cutaneous defects, reconstructive techniques are required to provide adequate coverage of the surgical bed in order to preserve the functionality of the affected region and achieve satisfactory aesthetic outcomes.

In this context, we present the case of a patient with LM localized to the left lower hemiabdomen, managed with wide surgical excision and immediate defect reconstruction using a pedicled anterolateral thigh (ALT) flap. Given the size of the defect and exposure of deep fascial structures, a regional pedicled ALT flap was selected to ensure reliable vascularized coverage without abdominal wall muscle sacrifice. This case highlights the usefulness of this technique in the reconstructive management of extensive cutaneous defects secondary to deep mycoses, owing to its versatility, reliable vascularity, and low donor site morbidity.

## Case report

**Figure 1.**
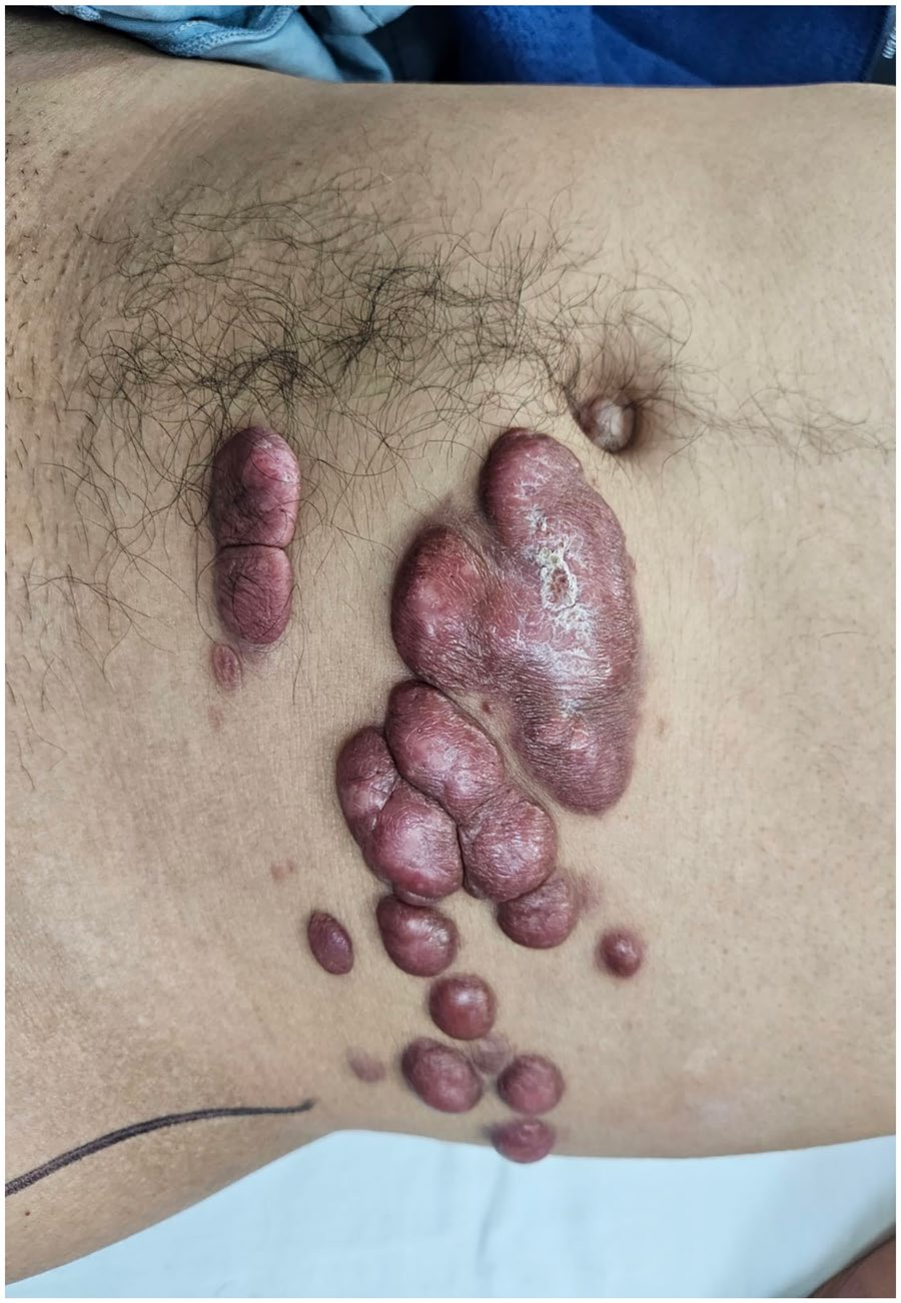
Initial clinical presentation showing multiple confluent hyperkeratotic nodular lesions on the left hemiabdomen, large in size with keloid-like appearance.

**Figure 2.**
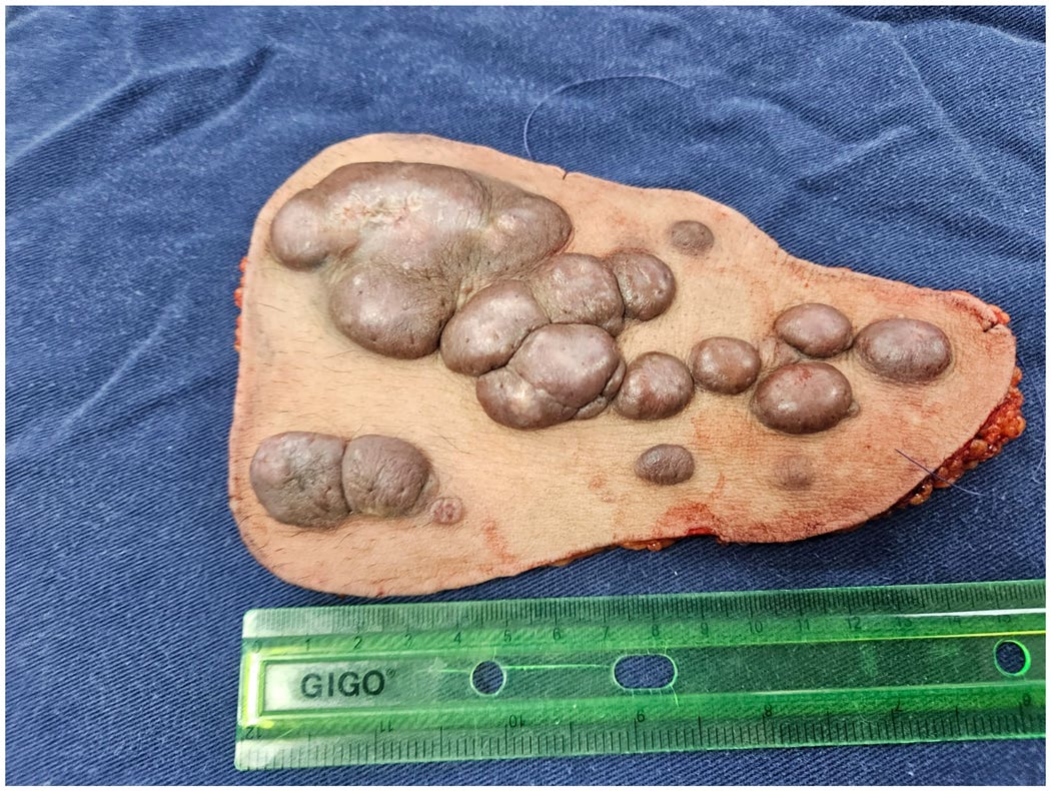
Complete surgical specimen submitted for histopathological examination.

**Figure 3.**
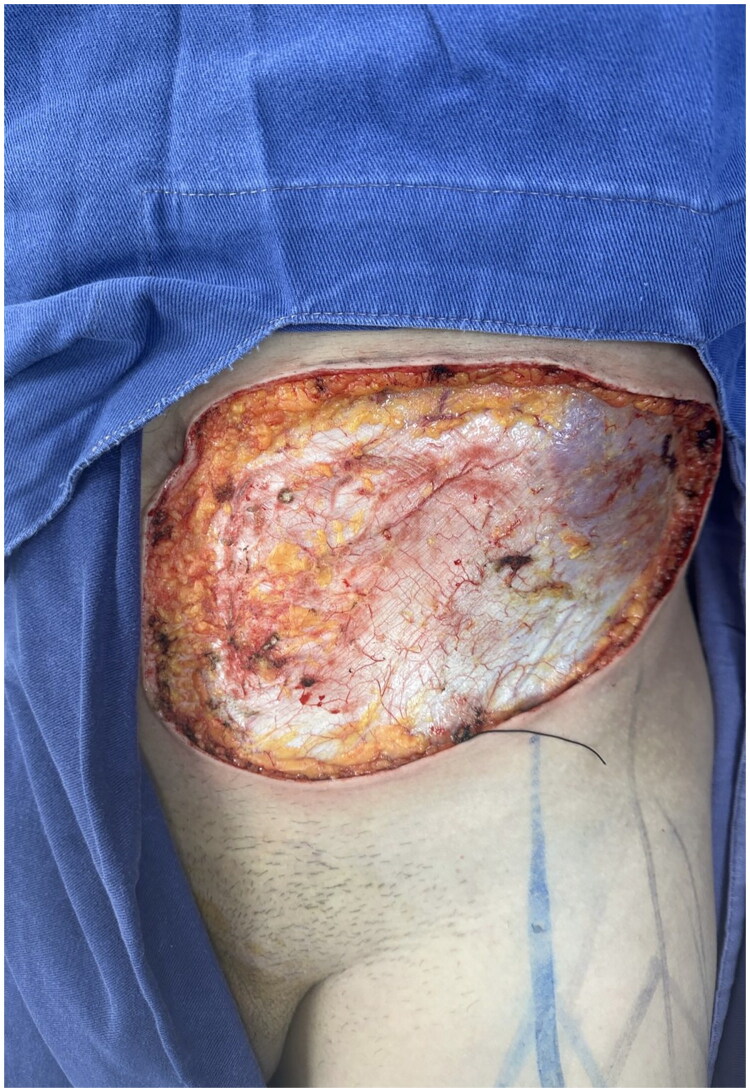
Extensive surgical defect resulting from wide excision of cutaneous and subcutaneous lesion, with exposure of deep planes and neurovascular structures, corresponding to advanced abdominal lobomycosis.

A 42-year-old male patient was referred from the dermatology department due to a three-year history of progressive, confluent, hyperkeratotic nodular lesions on the left hemiabdomen, measuring approximately 20 × 10 cm, consistent with LM (Figure 1). He underwent wide local excision with 2 cm macroscopic margins determined by the soft tissue surgery team based on the clinically apparent extension of the lesion and previously reported high recurrence rates following incomplete excision. Dissection was carried out through tissue planes down to the muscular fascia of the rectus abdominis and external oblique muscles, with en bloc excision of the affected segment, including skin, subcutaneous tissue, and muscular fascia (Figure 2). This resulted in a complex and extensive coverage defect, with exposure of the anterior rectus sheath and the aponeurosis of the external oblique muscle. No major named vascular trunks were exposed; however, the deep fascial layer was completely denuded (Figure 3).

Given the dimensions and characteristics of the defect, the plastic surgery team performed reconstruction with a pedicled anterolateral thigh (ALT) flap. Dissection was carried out through the tissue planes until identification of musculocutaneous perforator vessels arising from the descending branch of the lateral circumflex femoral artery. These perforators were carefully dissected through their intramuscular course within the vastus lateralis to obtain adequate pedicle length. To increase the arc of rotation and facilitate tension-free reach to the lower abdominal defect, the flap was rotated approximately 180 degrees in a cephalad direction based on its proximal pedicle. No microvascular anastomosis was performed, as the flap was transferred as an antegrade pedicled ALT flap.

**Figure 4.**
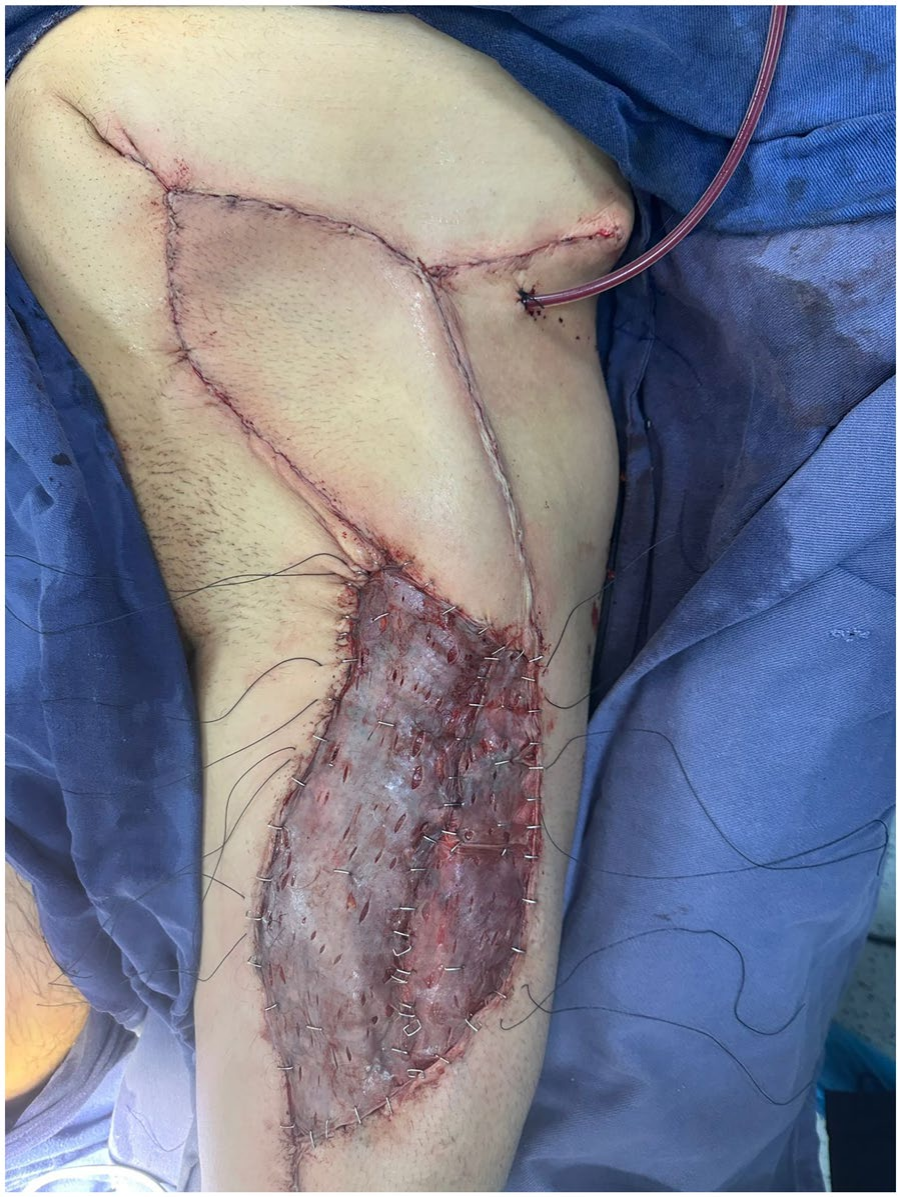
Intraoperative view of defect reconstruction with pedicled ALT flap; donor site reconstructed with split-thickness skin graft.

Adjacent abdominal skin was circumferentially undermined approximately 3–4 cm to facilitate tension-free inset. Layered closure was achieved, obliterating the subcutaneous dead space. Clinically, the flap demonstrated adequate viability without pedicle torsion or vascular compromise. At the donor site (right thigh), a split-thickness skin graft was harvested with an electric dermatome at an approximate depth of 0.012–0.015 inches, fixed with metal staples, and dressed with conventional quilting using furacine-impregnated gauze and cotton pads, followed by placement of a transparent dressing to promote a moist chamber environment (Figure 4).

Postoperatively, the patient received systemic antifungal therapy with itraconazole as adjuvant treatment, given the chronic nature of LM and the potential risk of recurrence despite complete surgical excision.

**Figure 5.**
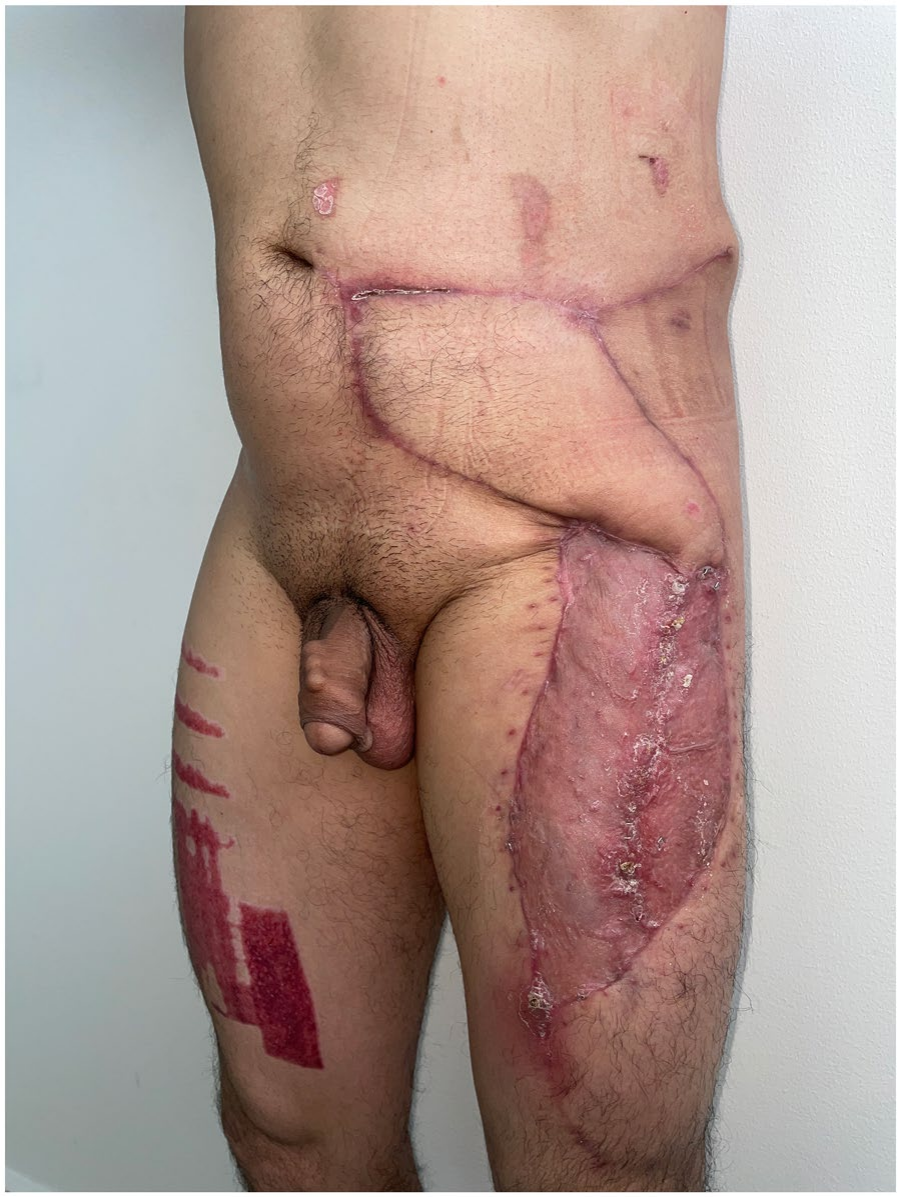
Postoperative outpatient evaluation: viable ALT flap without signs of compromise; donor site graft shows complete integration.

During outpatient follow-up, the flap remained viable without signs of arterial or venous compromise. The skin grafts showed complete integration, with no clinical evidence of local infection (Figure 5).

Histopathological examination revealed epidermis with areas of mild acanthosis and foci of pseudoepitheliomatous hyperplasia. The dermis showed a chronic inflammatory infiltrate composed of histiocytes, lymphocytes, and fibroblasts. Within the cytoplasm of histiocytes and in the adjacent stroma, multiple spherical yeasts measuring 6–12 μm in diameter were identified, with thick refractile walls, prominent capsules, and arranged in linear chains or ‘rosary-like’ patterns, a characteristic finding of *Lacazia loboi*. Special stains (PAS, PAS-D, and Grocott’s methenamine silver [GMS]) strongly highlighted the encapsulated chained yeasts, while Fontana–Masson staining revealed melanin pigment in some cells.

## Discussion

LM remains a poorly recognized deep mycosis, frequently underdiagnosed, particularly in non-endemic regions (Table 1). This underestimation is related to its slow and insidious course, its clinical similarity with other chronic dermatopathies, and the limited awareness among healthcare professionals regarding its distinctive clinical, histopathological, and microbiological features.

**Table 1.** Reported cases of lobomycosis in the literature.

Article	Authors	Year	Country	Population	Lesion	Treatment	Antifungal	Recurrence
Human Case of Lobomycosis	Elsayed et al. [1]	2004	Canada	1 patient, 42 years, female	Plaque-like lesion with keloid scar on posterior right arm	Complete surgical excision	None	No
Lobomycosis: a case from Southeastern Europe and review of the literature	Papadavid et al. [2]	2012	Greece	1 patient, 64 years, female	Erythematous infiltrated plaque, 3 cm, keloid-like, inner left thigh	No excision due to systemic deterioration	None	N/A (died of sepsis)
Jorge Lobo’s disease: a case of keloidal blastomycosis (lobomycosis) in a nonendemic area	Arju et al. [3]	2014	USA	1 patient, 65 years, male	Multiple keloid-like nodules on arms and right elbow	Local surgeries (multiple excisions)	Not specified; no improvement	Yes (new lesions)
Lobomycosis: epidemiology, clinical presentation and management	Francesconi et al. [4]	2014	Brazil	Review (249 cases)	Multiple keloid-like lesions on exposed areas; chronic, slow progression; dissemination by repeated trauma or self-inoculation	Surgery for early lesions; combinations: surgery + antifungals	Posaconazole, Itraconazole, Clofazimine	High recurrence rate expected
Lobomycosis of the Lower Limb in an Amazonian Patient	de Souza et al. [5]	2015	Brazil	1 patient, 56 years, male	Multiple ulcerated, pruritic, bleeding keloid nodules on left leg after trauma with plant spine	Surgical excision	Posaconazole	Not reported
Leprosy and Lobomycosis: First report from the Amazon Region	Ihara et al. [6]	2015	Brazil	1 patient, 89 years, male	Keloid lesion of 30 years on left clavicle	Patient refused treatment	None	–
Lobomycosis: a therapeutic challenge	Araújo et al. [7]	2018	Brazil	1 patient, 36 years, male	Infiltrative keloid nodules on left ear (lobule and posterior helix)	Wide excision + cryotherapy every 3 months + follow-up	–	–
Lobomycosis in Soldiers, Colombia	Arenas et al. [8]	2019	Colombia	6 patients, 24–41 years, female	Keloid-like nodular lesions on leg, sternum, finger, face, arm, forearm	Surgical excision; some with Itraconazole + Clofazimine	Itraconazole 100 mg/d ± Clofazimine	1 recurrence
Plastic surgery for the treatment of contagious diseases: lobomycosis	Korte et al. [9]	2019	Brazil	22 patients (mostly men, ∼50 years)	Monomorphic lesions (keloid nodules) and polymorphic (plaques, verrucous, ulcerated) on ears, limbs, face, chest	Surgery in all patients, most with antifungals; 8-year follow-up	Not specified (generally itraconazole)	Yes (9 of 11 patients)
Invasive Basidiobolomycosis Presenting as Retroperitoneal Fibrosis	Alsharidah et al. [10]	2020	Saudi Arabia	1 patient, 39 years, female	Mass encasing aorta, mesenteric artery, and renal vein in retroperitoneum	No surgery	Itraconazole 200 mg BID	No
Lobomycosis: exuberant presentation with malignant transformation	Lima et al. [11]	2021	Brazil	1 patient, 83 years, male	Multiple keloid nodules on limbs; large ulcerated tumor in left cubital fossa with tendon exposure; 30-year evolution	Palliative care (due to associated squamous cell carcinoma)	Not reported	N/A (died)
Human Lobomycosis Caused by Paracoccidioides (Lacazia) loboi, Panama, 2022	Suárez et al. [12]	2023	Panama	1 patient, 87 years, female	Multiple indurated, hyperpigmented nodules from thigh to foot on left leg	Itraconazole 200 mg/day; cryotherapy pending	Itraconazole	Partial improvement; persistent lesions
Squamous cell carcinoma of auricular pavilion in patient with Jorge Lobo’s disease: case report	Castro et al. [13]	2024	Brazil	1 patient, 63 years, male	Ulcerated, keloid lesions in right auricular region	Resection + neck dissection + pectoralis major flap	Not mentioned	Yes (malignant degeneration)
Gastrointestinal Basidiobolomycosis: A Case Series	Meeralam et al. [14]	2024	Saudi Arabia	5 patients, 23–54 years, 3 males/2 females	Infiltrative masses and inflammatory lesions in colon, rectum, liver, ileum, sigmoid	Hemicolectomy, total colectomy, partial hepatectomy	Itraconazole or Voriconazole for 18 months	None (short follow-up)
Lobomycosis: single lesion on the lip	Nunes et al. [15]	2024	Brazil	1 patient, 66 years, male, farmer	Infiltrative, erythematous, firm tumor on right upper lip; 10-year evolution	Surgical excision with margins + itraconazole 100 mg q12h × 6 months	Itraconazole	No
Lobomycosis: three atypical cases in Colombia	Carbonell-García et al. [16]	2024	Colombia	3 patients: 2 men (29 and 42 years), 1 woman (44 years)	Keloid-like nodular lesions on face, ear, and arm; shiny, ulcerated, or crusted	Surgery in all; one with plastic surgery involvement	Itraconazole in 1 case	Yes (historical recurrence in 2 cases)
Lobomycosis in Amazon Region, Bolivia, 2022	Méndez et al. [17]	2024	Bolivia	1 patient, 71 years, male	Infiltrated nodular keloid plaque involving entire left ear	Surgical excision	Itraconazole 200 mg/day × 3 months (discontinued due to side effects)	Yes

From a therapeutic perspective, wide surgical excision remains the treatment of choice, since recurrence rates between 30% and 50% have been reported in cases of incomplete resection [3,6,7]. This underscores the need for adequate safety margins to achieve effective local disease control. In the absence of standardized margin guidelines, resection width is generally determined by macroscopic disease extension and clinical judgment, aiming to minimize the risk of local recurrence.

Regarding reconstruction of large defects resulting from wide resections, especially those located in complex regions such as the lower abdomen and inguinal area, the selection of reconstructive technique should be based on criteria that ensure safe tissue coverage, functional restoration, and satisfactory aesthetic outcomes. In this context, pedicled flaps represent an effective and reliable option. Among them, the vertical rectus abdominis myocutaneous (VRAM) flap [18], the tensor fasciae latae (TFL) flap, and free-style perforator flaps are noteworthy [19]. However, in cases requiring large tissue volumes or in recipient beds with unfavorable conditions such as fibrosis, prior radiation, or multiple surgeries, free flaps emerge as a versatile and reliable alternative, including the deep inferior epigastric perforator (DIEP) flap and ALT flap [20].

The choice among these options must be individualized, considering multiple variables such as the size, shape, and location of the defect, the quality of surrounding tissues, the patient’s general condition, comorbidities, and history of local or systemic treatments. In the present case, the defect was extensive and complex, located in the lower left abdomen, which rendered both primary closure and split-thickness skin grafting unfeasible. Therefore, a pedicled ALT flap was selected. This decision was supported by the flap’s robust vascular supply and long pedicle, based on septocutaneous or musculocutaneous perforators of the descending branch of the lateral circumflex femoral artery, allowing regional transfer without the need for microvascular anastomosis [20,21]. In our case, musculocutaneous perforators were dissected through the vastus lateralis to obtain sufficient pedicle length, and a 180-degree rotation was performed to increase the arc of reach to the lower abdominal defect without vascular compromise. In addition, its variability in tissue composition and low donor-site morbidity consolidate it as a fundamental tool in the reconstructive surgeon’s armamentarium [22,23]. Compared to VRAM flaps, the ALT flap avoids sacrifice of abdominal wall musculature, thereby preserving core function and potentially reducing donor-site morbidity.

Finally, a systematic review of the literature from the past 25 years revealed that most reported cases initially received systemic antifungal therapy. However, due to the absence of adequate clinical response, surgical resection was ultimately adopted as the definitive therapeutic measure. Despite these approaches, considerable heterogeneity persists in the management of LM, and currently no standardized consensus exists, underscoring the need for continued documentation of clinical experiences to generate evidence-based recommendations. Furthermore, the literature reports very few cases of complex reconstructions secondary to LM, highlighting the relevance of the present case as a significant contribution to the clinical and surgical understanding of this rare entity. In our patient, adjuvant systemic antifungal therapy was administered postoperatively as a complementary strategy, although surgical excision remains the cornerstone of definitive management.

## Conclusion

Advanced lobomycosis can result in complex defects requiring equally complex reconstructive solutions following wide surgical excision. The pedicled ALT flap constitutes a reliable alternative in plastic surgery due to its versatility, robust vascularity, and low donor-site morbidity, allowing safe coverage of exposed deep fascial structures and satisfactory functional and aesthetic outcomes in challenging anatomical regions. Careful perforator dissection and adequate arc of rotation, including 180-degree transfer when necessary, enable tension-free inset without the need for microvascular anastomosis.
